# Supplementary material for: CDK4 inactivation inhibits apoptosis via mitochondria-ER contact remodeling in triple-negative breast cancer
Source: Nat Commun. 2025 Jan 9;16:541. doi: 10.1038/s41467-024-55605-z (PMC11718081; doi:10.1038/s41467-024-55605-z)
Supplement: Supplementary file 1 — Supplementary Information [file 41467_2024_55605_MOESM1_ESM.pdf]

Supplementary Figure 1

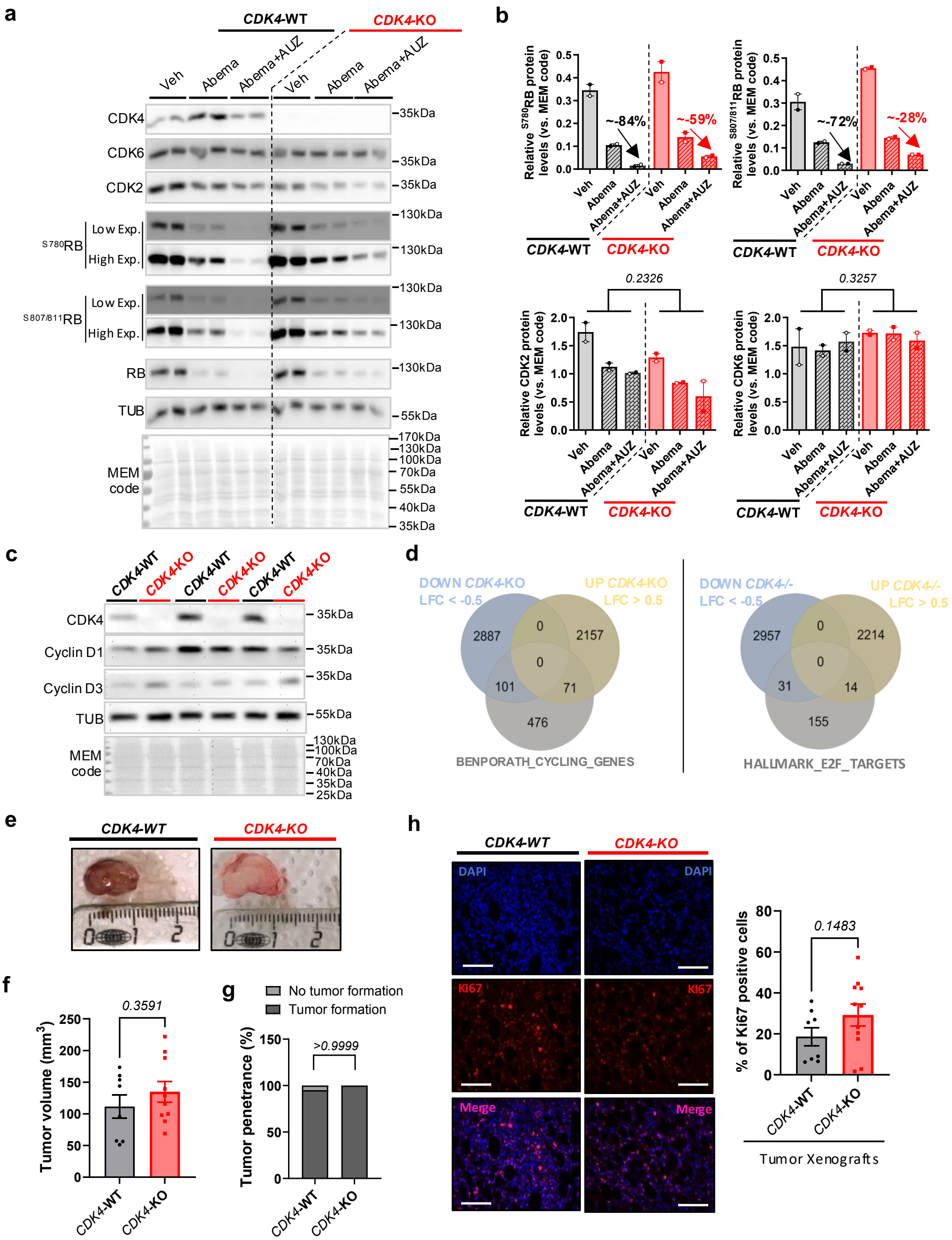

Supplementary Figure 2

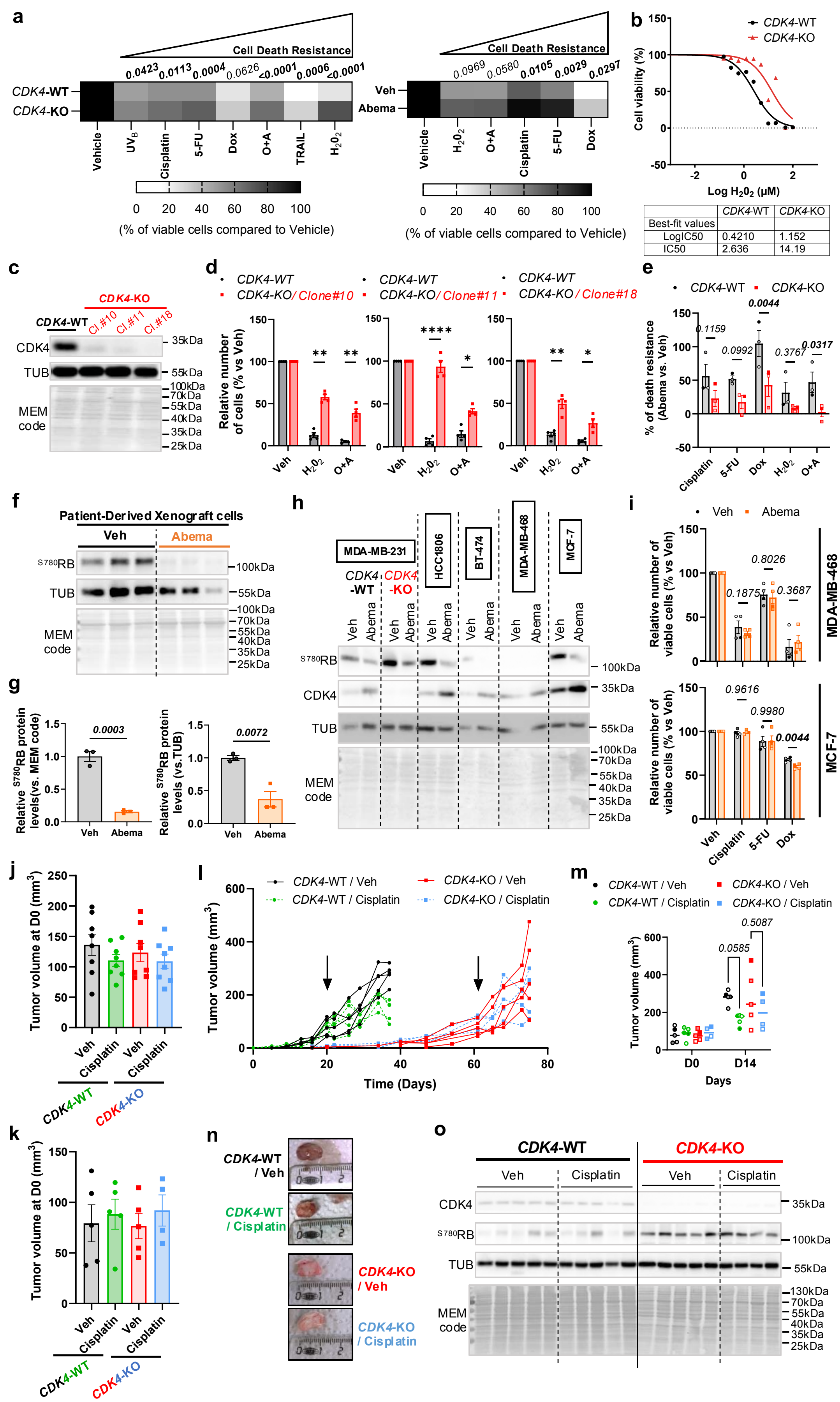

Supplementary Figure 3

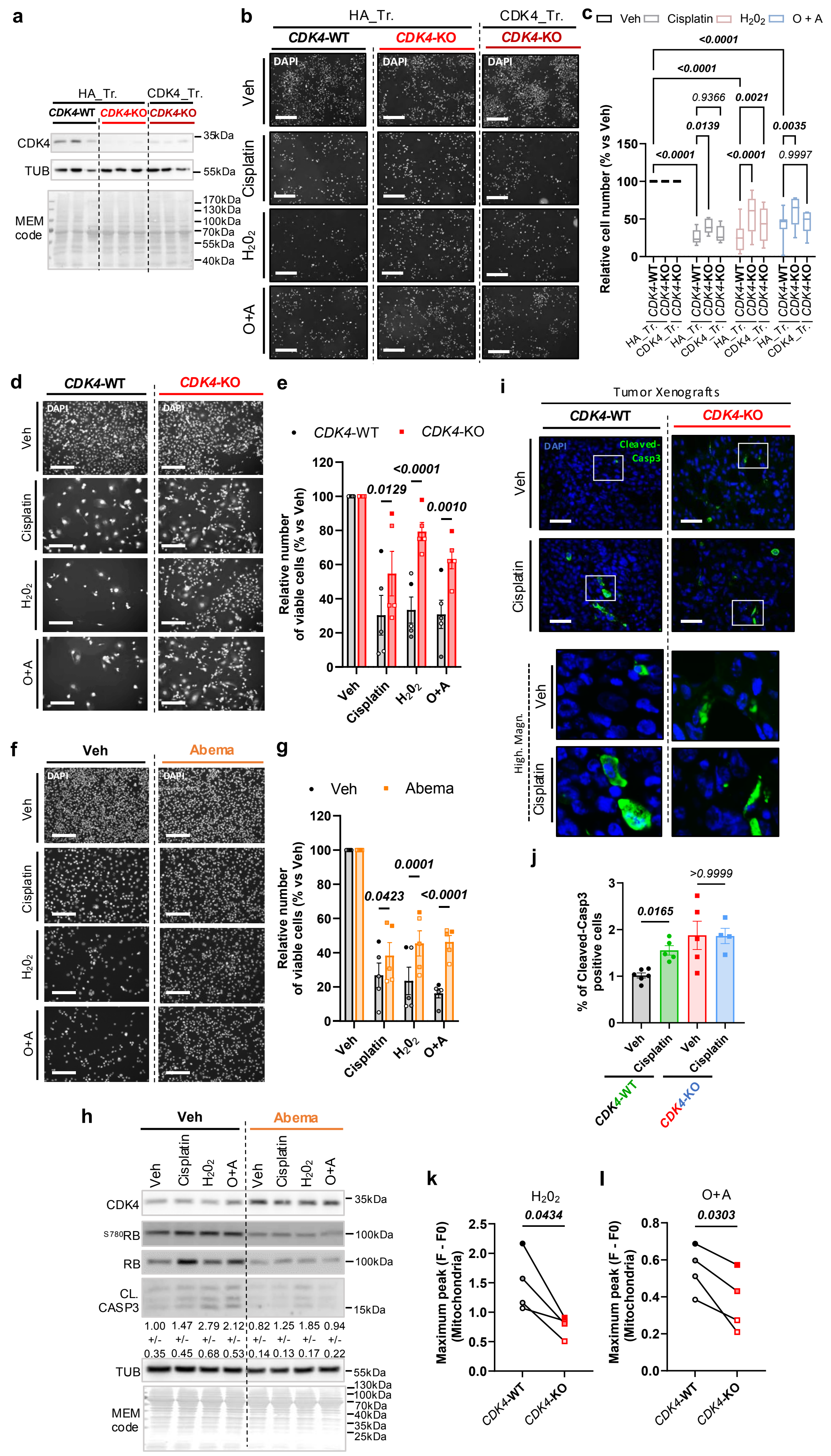

Supplementary Figure 4

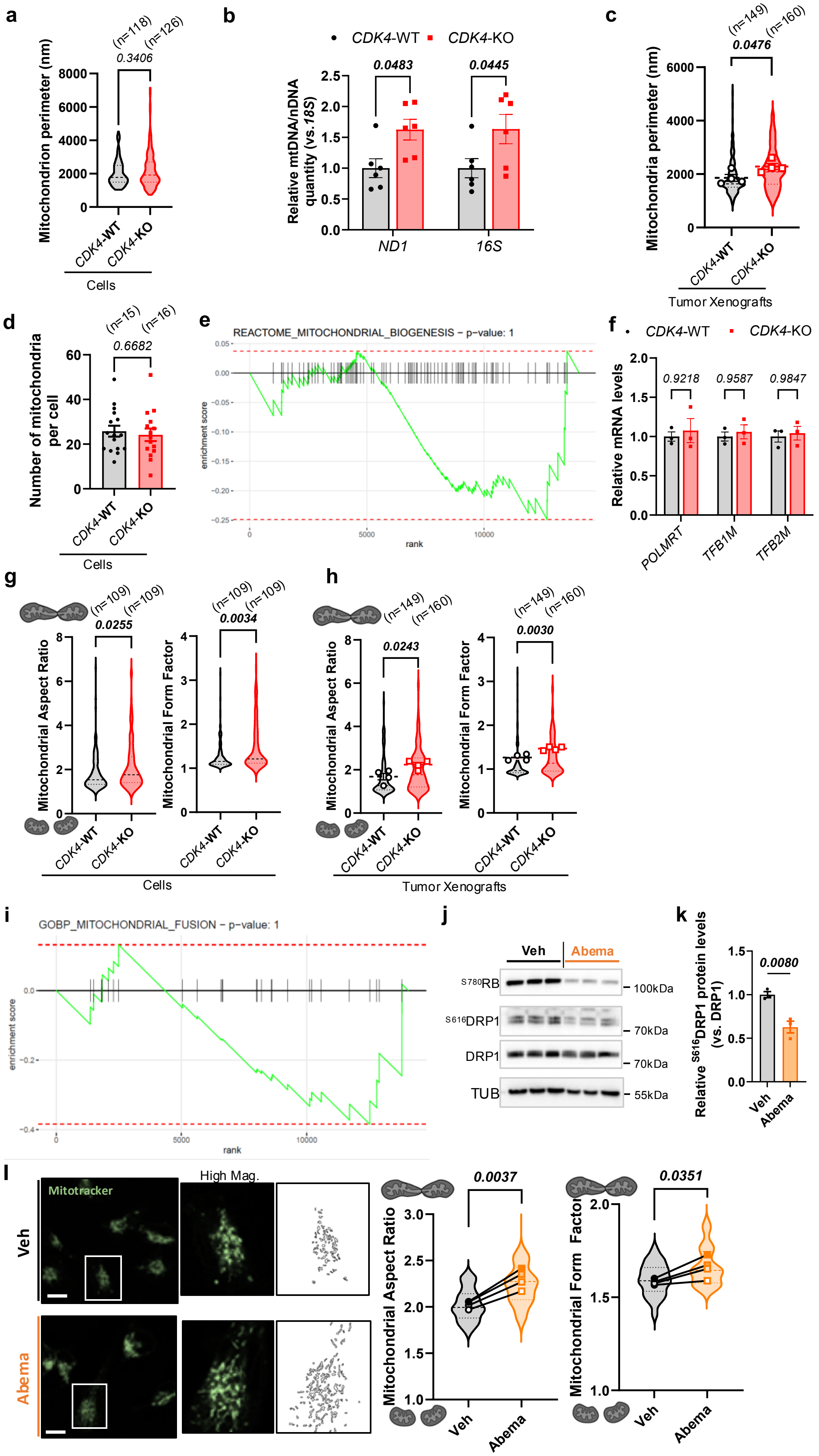

Supplementary Figure 5

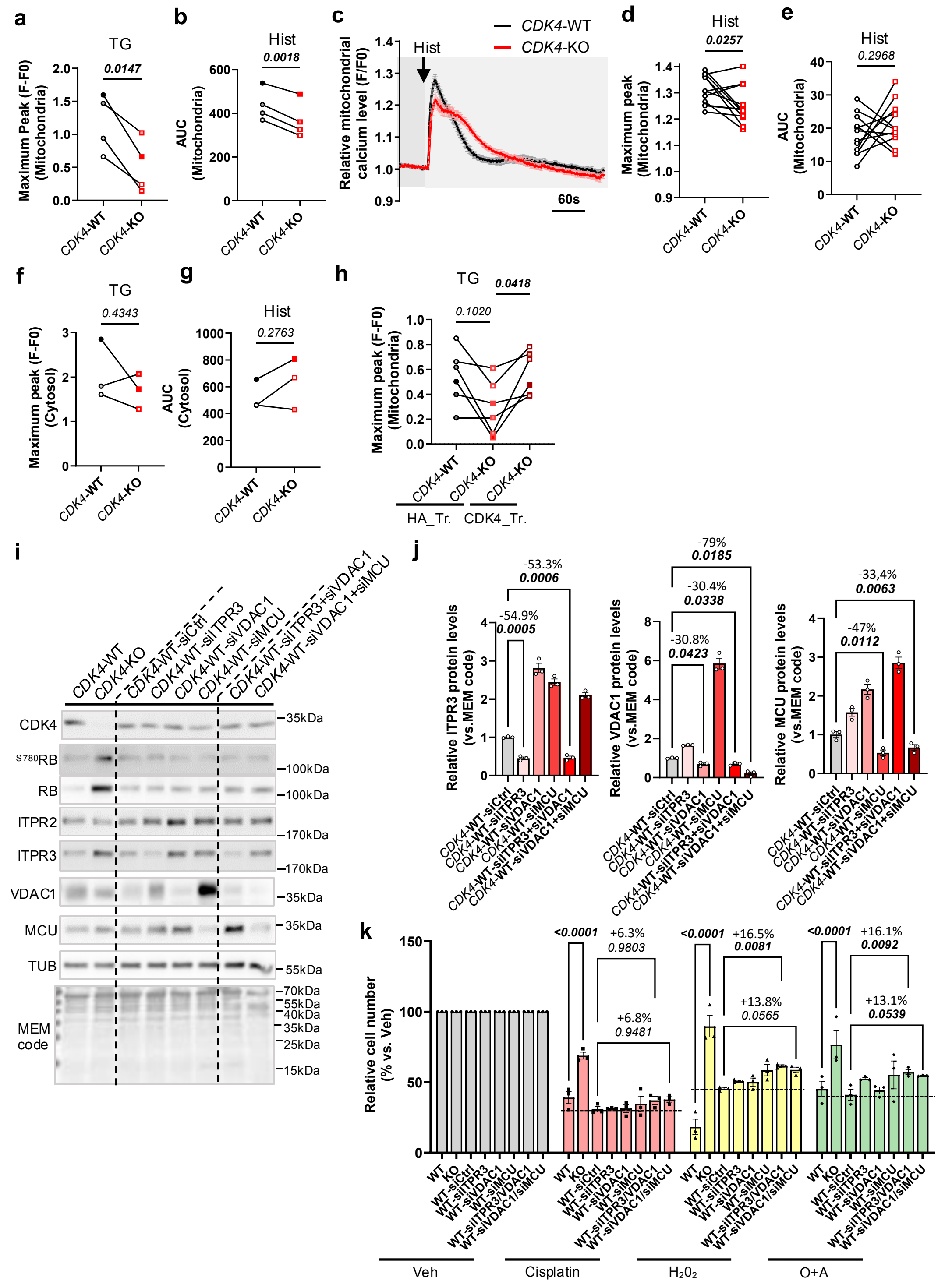

Supplementary Figure 6

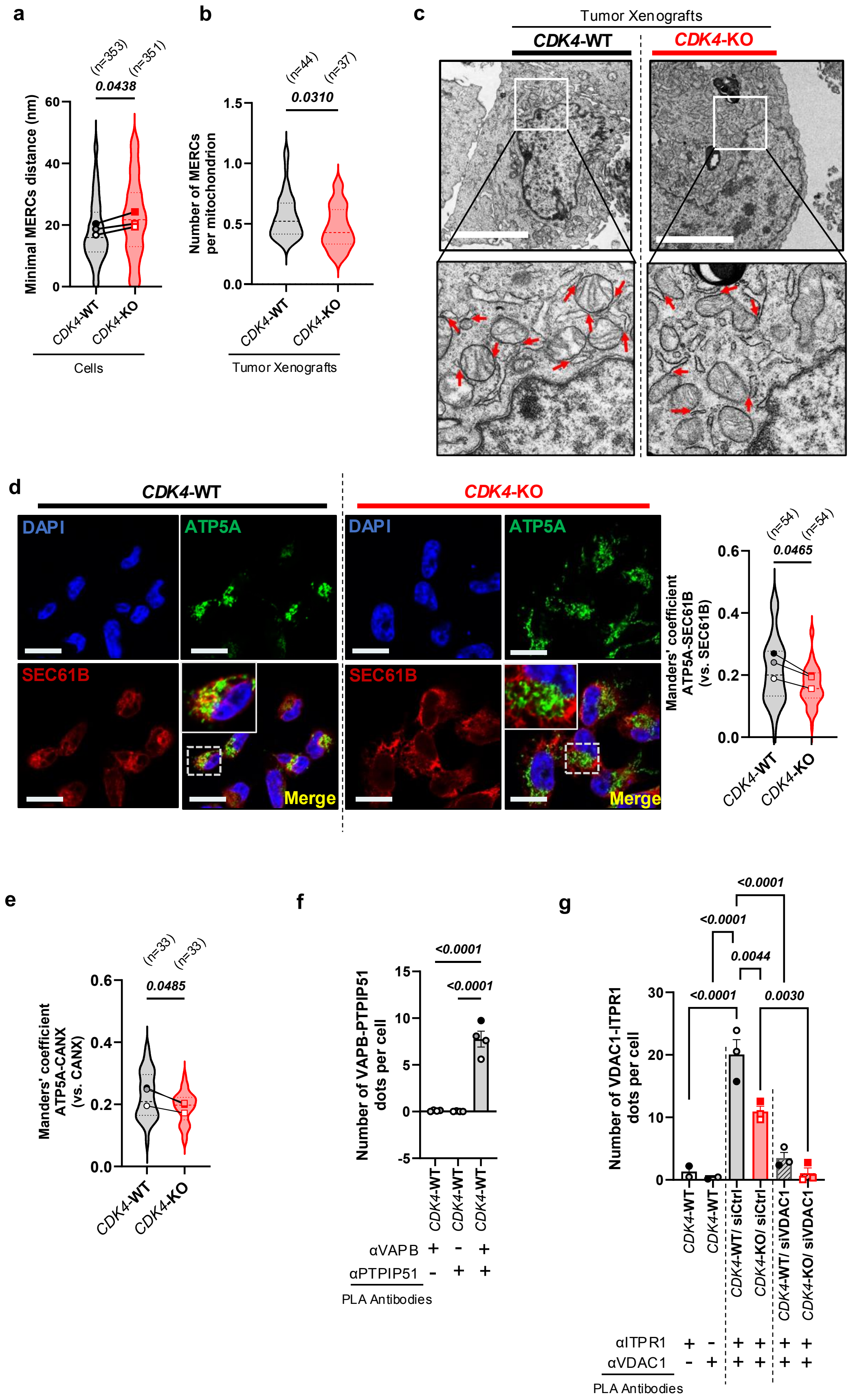

Supplementary Figure 7

a

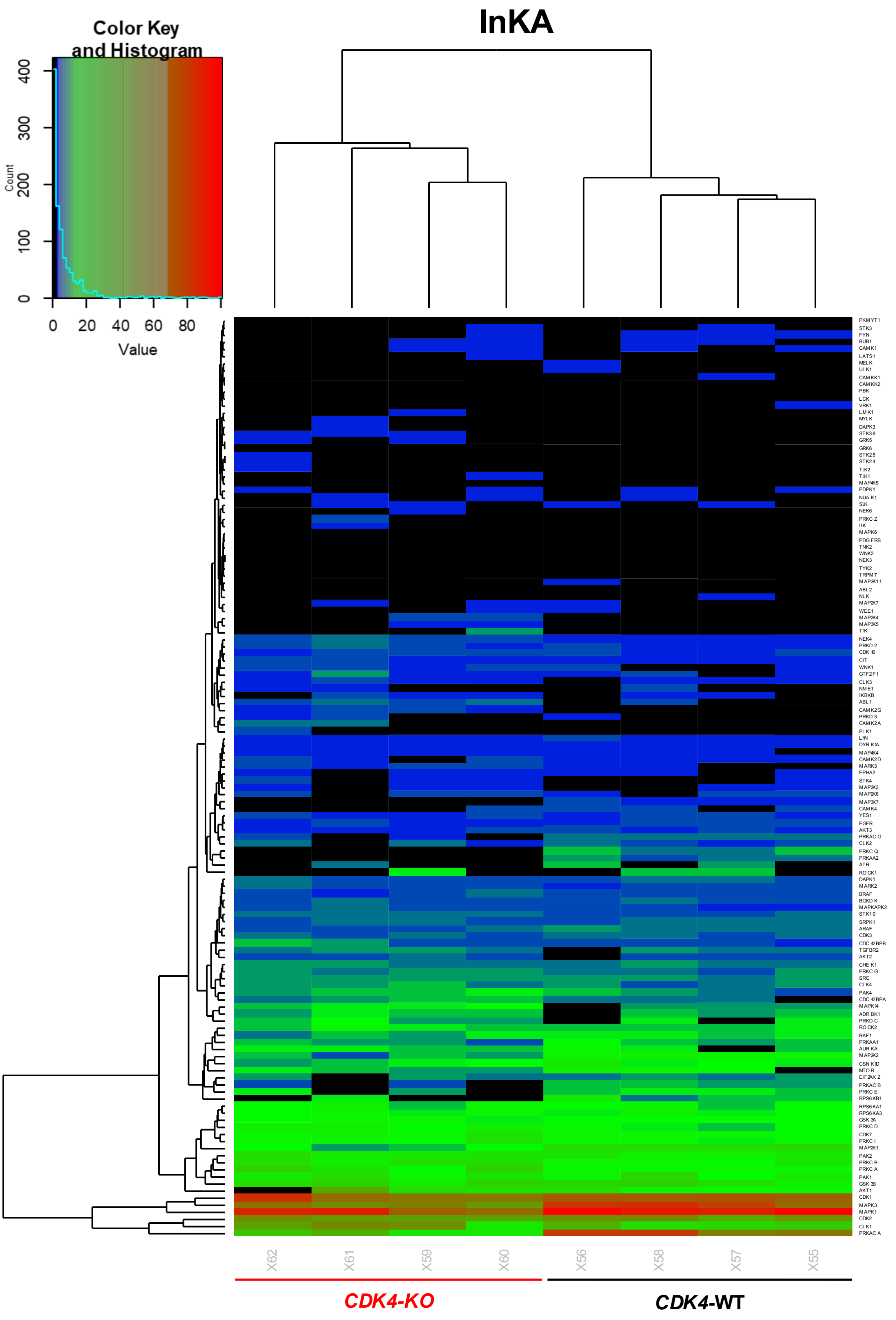

Supplementary Figure 7

b

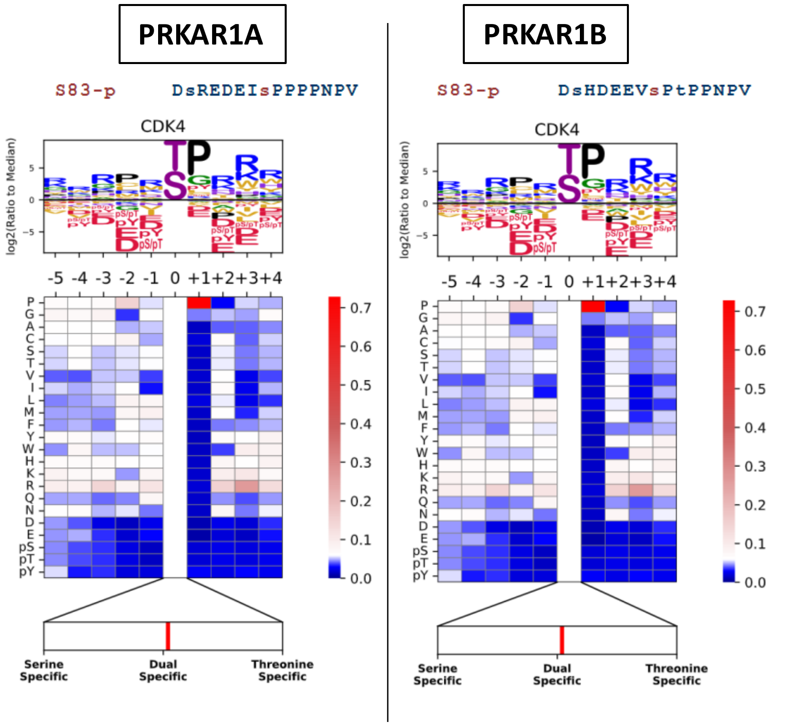

c

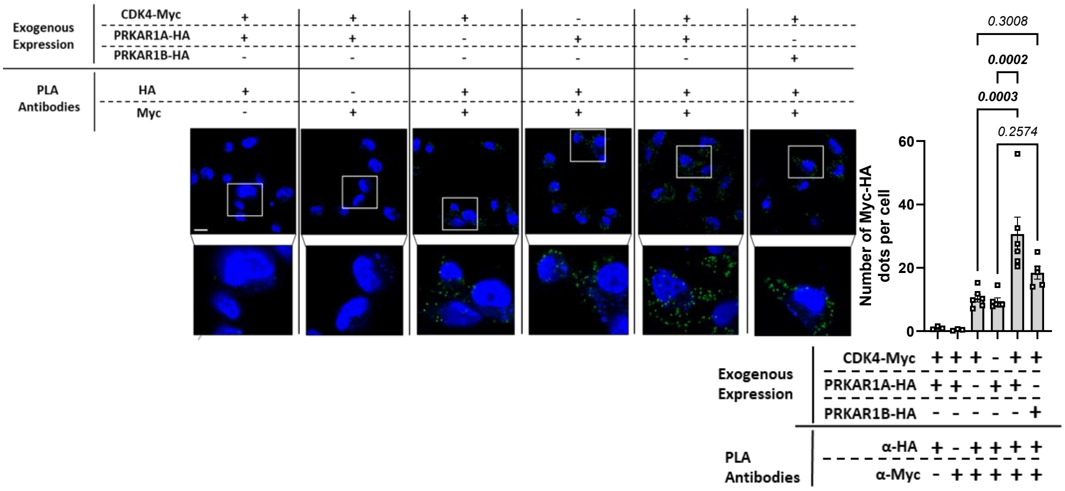

Supplementary Figure 8

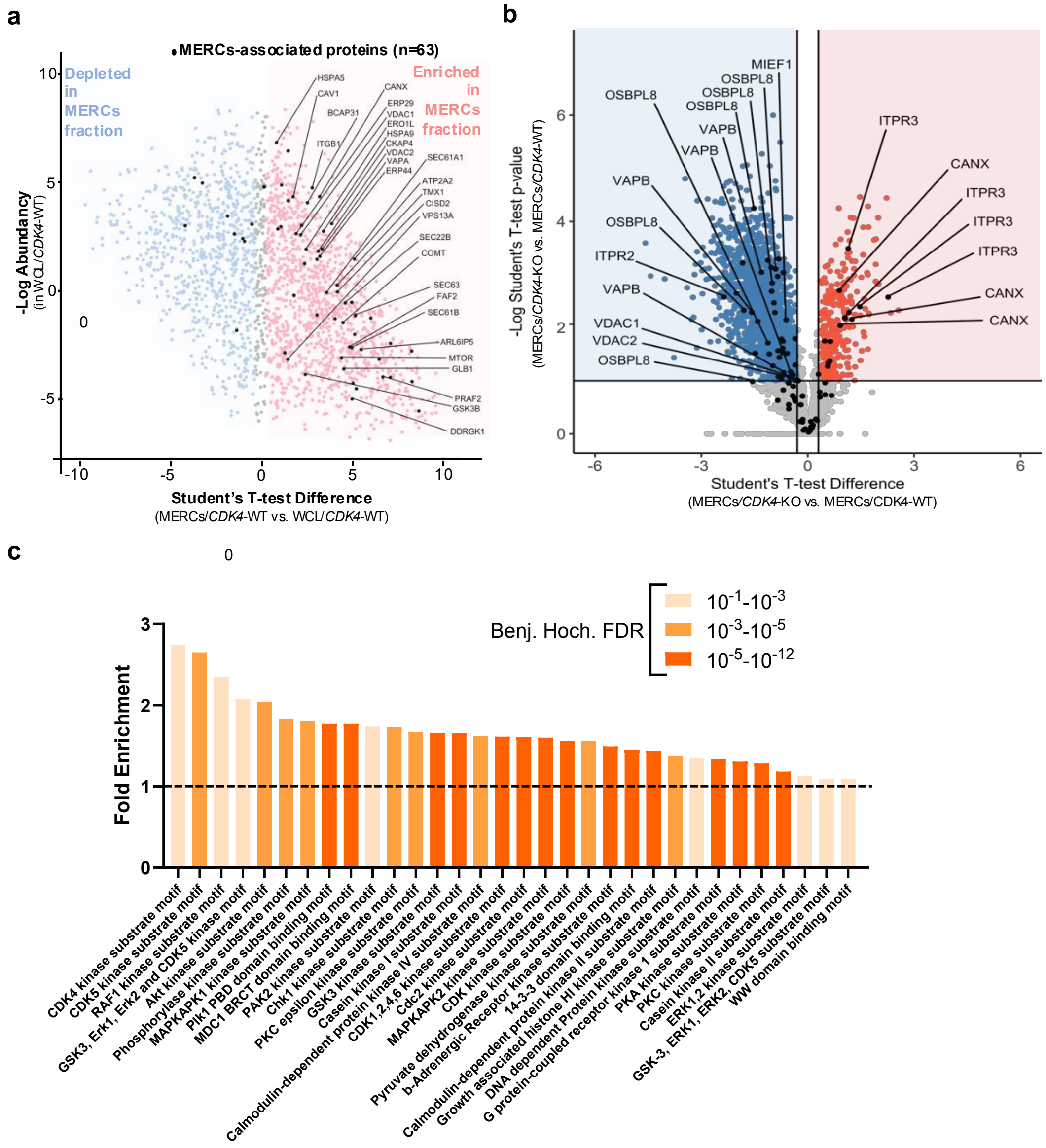

Supplementary Figure 9

a

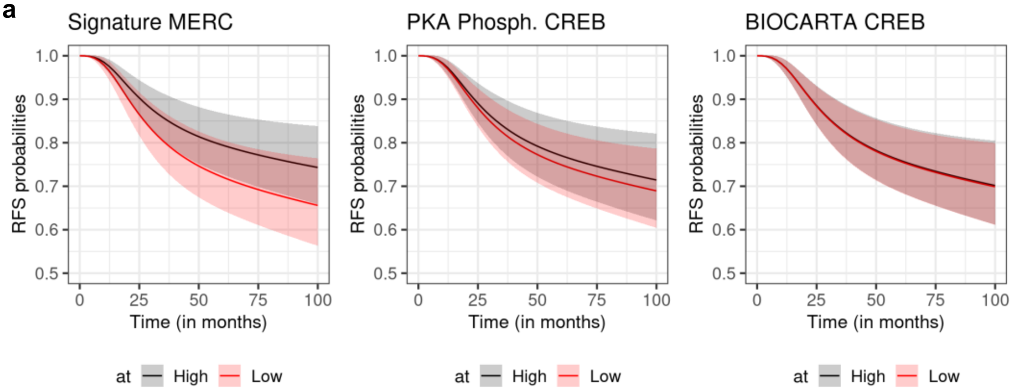

Supplementary Figure 10

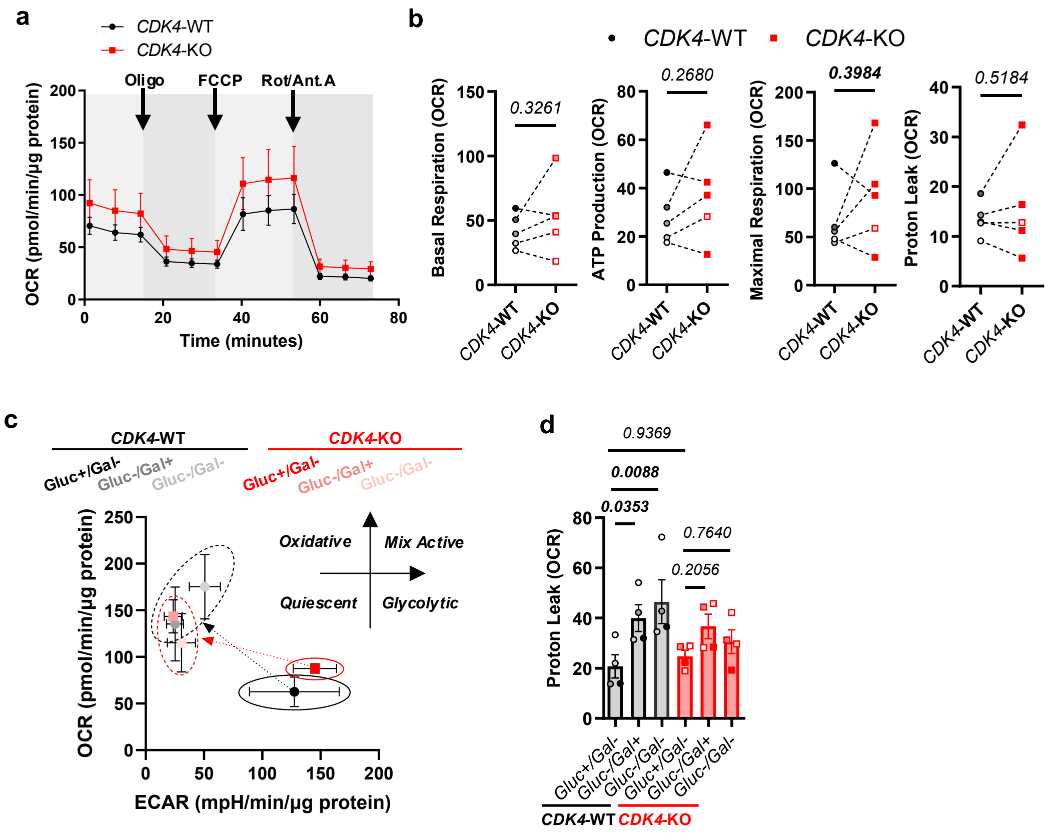

Supplementary Figure 11

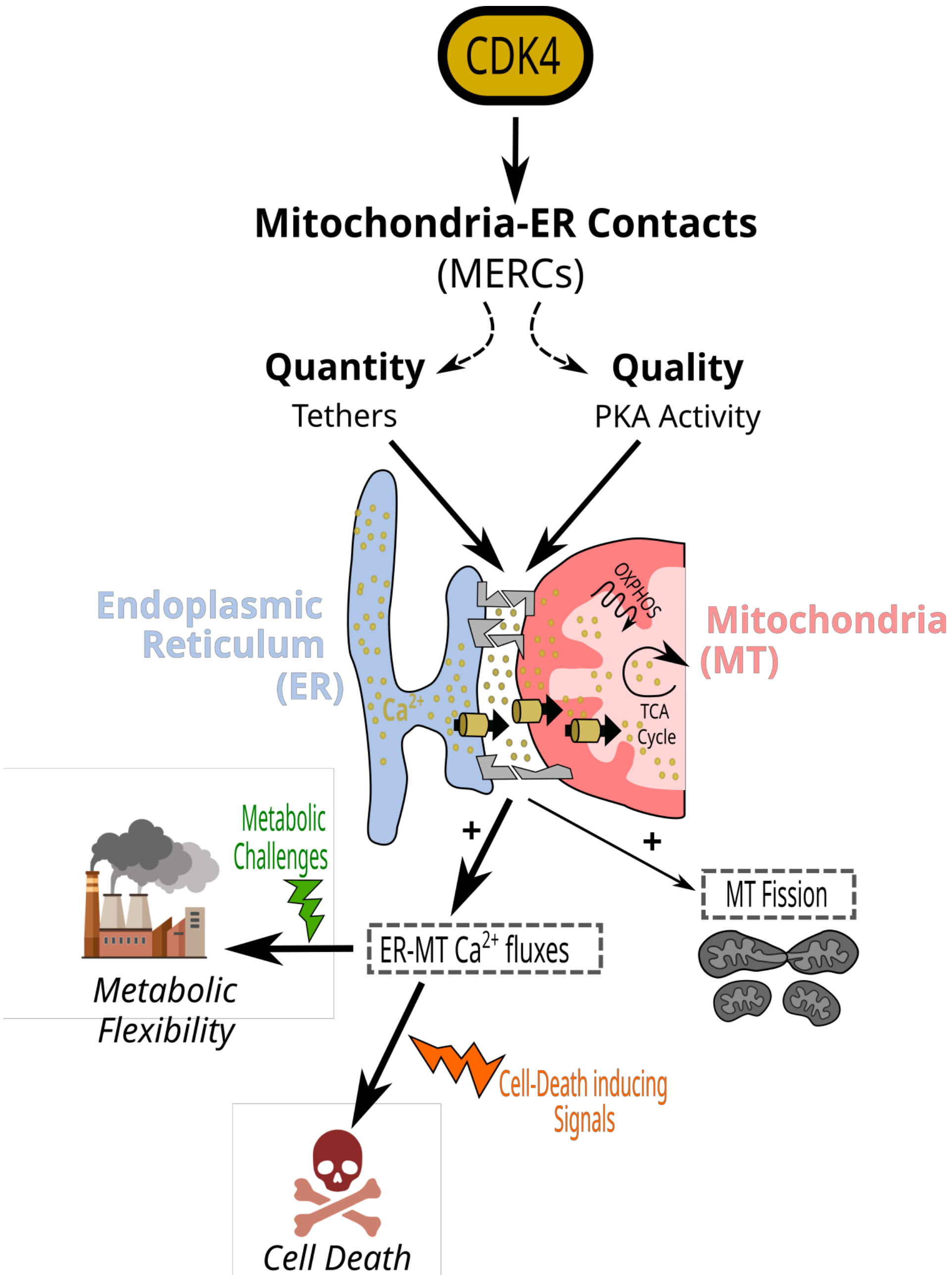

Supplementary Table 1

ACSL4	BECN1	EIF2AK3	HSP90B1	MTOR	PPID	SACM1L	TARDBP
AHCYL1	BOK	ERN1	HSPA5	MTX2	PPP2CA	SAR1A	TBL2
AKT1	BRCA1	ERO1L	HSPA9	NAPG	PPP2R4	SAR1B	TCHP
AKT2	CABP1	ERP29	INF2	NCS1	PRAF2	SCCPDH	TDRKH
AKT3	CALR	ERP44	ITGB1	NDUFA8	PRRX1	SEC22B	TESPA1
ANXA7	CANX	EXD2	ITPR1	NDUFB6	PSEN1	SEC61A1	TGM2
APP	CAV1	FAF2	ITPR2	NLRP3	PSEN2	SEC61B	TMX1
ARL6IP5	CCDC47	FATE1	ITPR3	NOX4	PTEN	SEC63	TOMM70A
ARMCX3	CDIP1	FIS1	KRAS	OCIAD1	PTRH2	SEPN1	TP53
ATAD3A	CIB1	FKBP10	LRRC59	OSBPL5	PYCARD	SHC1	TSPO
ATF6	CISD2	FKBP8	MAVS	OSBPL8	RAB1A	SIGMAR1	TXNIP
ATG14	CKAP4	FUNDC1	MCL1	PACS2	REEP1	SLC25A4	UBXN4
ATP2A1	CLCC1	FUNDC2	MCU	PARK2	RHOT1	SLC25A5	VAPA
ATP2A2	COMT	FUS	MCUR1	PARK7	RICTOR	SLC25A6	VAPB
ATP2A3	CPD	GDAP1	MFF	PDIA3	RMDN3	SNCA	VDAC1
BAK1	CYB5R3	GLB1	MFN1	PDK4	RPS7	SOD1	VDAC2
BAP1	CYC1	GSK3B	MFN2	PDZD8	RTN1	SRPRB	VDAC3
BAX	CYCS	HK2	MICU1	PGRMC1	RTN4	STARD1	VPS13A
BCAP31	DDRGK1	HMOX2	MICU2	PGRMC2	RYR1	STAT3	VPS13C
BCL2	DHCR7	HNRNPR	MIEF1	PINK1	RYR2	STT3B	VPS13D
BCL2L1	DNM1L	HRAS	MIEF2	PML	RYR3	STX17	WAVE3
BCL2L13	EEF1D	HSD17B12	MOSPD2	PPARGC1A	S100A1	SYNJ2BP	WFS1

Supplementary Table 2

Gene Name	Main-associated Functions			Gene Name	Main-associated Functions		
ACSL4	Lipids			MICU1	Ions Transport		
AHCYL1	Others			MICU2	Ions Transport		
AKT1	Oncogenes			MIEF1	MT Dynamics		
AKT2	Oncogenes			MIEF2	MT Dynamics		
AKT3	Oncogenes			MOSPD2	Tethering	Lipids	
ANXA7	Ions Transport			MTOR	Autophagy	Signalling	
APP	Others			MTX2	MT Bioenergetics		
ARL6IP5	MT Bioenergetics			NAPG	ER Homeostasis		
ARMCX3	MT Dynamics			NDUFA8	MT Bioenergetics		
ATAD3A	Tethering			OCIAD1	MT Bioenergetics		
ATF6	ER Homeostasis			OSBPL5	Tethering	Lipids	
ATG14	Autophagy			OSBPL8	Tethering	Lipids	
ATP2A2	Ions Transport			PARK7	MT Bioenergetics		
BAK1	Apoptosis			PDZD8	Tethering		
BAP1	Apoptosis			PGRMC1	Ions Transport		
BAX	Apoptosis			PGRMC2	Others		
BCAP31	Tethering			PML	Apoptosis		
BCL2	Apoptosis			PPID	Tethering		
BCL2L1	Apoptosis			PPP2CA	Signalling		
BCL2L13	Apoptosis			PPP2R4	Signalling		
BECN1	Apoptosis			PRAF2	Others		
CALR	ER Homeostasis	Ions Transport		PSEN1	Tethering		
CANX	Ions Transport			PTEN	Oncogenes		
CAV1	Ions Transport			PTRH2	Apoptosis		
CCDC47	Ions Transport			PYCARD	Apoptosis		
CIB1	ER Homeostasis	Ions Transport		RAB1A	ER Homeostasis		
CISD2	Tethering			RHOT1	Tethering		
CKAP4	Others			RICTOR	Autophagy	Signalling	
CLCC1	Ions Transport			RMDN3	Tethering		
COMT	MT Bioenergetics			RPS7	ER Homeostasis		
CPD	Others			RTN4	ER Homeostasis		
CYB5R3	MT Bioenergetics			RYR3	Ions Transport		
CYC1	MT Bioenergetics			SACM1L	ER Homeostasis	Lipids	
CYCS	MT Bioenergetics	Apoptosis		SAR1A	ER Homeostasis		
DDRGK1	ER Homeostasis			SAR1B	Others		
DHCR7	Lipids			SCCPDH	Others		
DNM1L	MT Dynamics			SEC22B	ER Homeostasis		
EEF1D	Others			SEC61A1	ER Homeostasis	Ions Transport	
EIF2AK3	ER Homeostasis	Lipids	Tethering	SEC61B	ER Homeostasis		
ERO1L	ER Homeostasis			SEC63	ER Homeostasis		
ERP29	ER Homeostasis			SHC1	Signalling		
ERP44	ER Homeostasis	Ions Transport		SIGMAR1	Tethering		
EXD2	MT Bioenergetics			SLC25A4	MT Bioenergetics		
FAF2	ER Homeostasis			SLC25A5	MT Bioenergetics		
FIS1	Tethering			SLC25A6	MT Bioenergetics		
FKBP8	Tethering			SRPRB	ER Homeostasis		
FUNDC2	Autophagy	MT Dynamics		STT3B	ER Homeostasis		
FUS	Tethering			STX17	Autophagy	Tethering	
GLB1	Autophagy			SYNJ2BP	Tethering		
GSK3B	MT Bioenergetics			TARDBP	Ions Transport		
HK2	MT Bioenergetics			TBL2	ER Homeostasis		
HMOX2	ER Homeostasis			TCHP	Tethering		
HNRNPR	Others			TDRKH	Others		
HSD17B12	Lipids	MT Bioenergetics		TGM2	Ions Transport		
HSP90B1	ER Homeostasis			TMX1	ER Homeostasis	MT Bioenergetics	
HSPA5	ER Homeostasis	Tethering		TOMM70A	MT Dynamics		
HSPA9	Tethering			TP53	Signalling	Apoptosis	
INF2	MT Dynamics			TSPO	Lipids		
ITGB1	Signalling			TXNIP	Signalling		
ITPR2	Ions Transport	Tethering		UBXN4	ER Homeostasis		
ITPR3	Ions Transport			VAPA	Tethering		
KRAS	Oncogenes			VAPB	Tethering		
LRRC59	Others			VDAC1	Ions Transport		
MAVS	Signalling			VDAC2	Ions Transport		
MCL1	Apoptosis			VDAC3	Ions Transport		
MCU	Ions Transport			VPS13A	Lipids		
MCUR1	Ions Transport			VPS13C	Lipids		
MFF	MT Dynamics			VPS13D	Tethering	Lipids	
MFN1	MT Dynamics	Tethering		WFS1	ER Homeostasis	Ions Transport	Tethering
MFN2	MT Dynamics	Tethering					

MT : Mitochondria; ER : Endoplasmic Reticulum

Supplementary Table 3

term	type_analysis	HR	conf.low	conf.high	p.value
CCND1	RFS	1.09	0.72	1.65	0.688
CCND2	RFS	0.78	0.61	0.99	0.045
CCND3	RFS	0.95	0.52	1.73	0.857
Signature CCNDs	RFS	0.89	0.74	1.06	0.178
CDK4	RFS	1.48	0.69	3.15	0.314
MERC	RFS	1.01	0.98	1.03	0.610
PKA Phosph. CREB	RFS	0.99	0.91	1.07	0.785
BIOCARTA CREB	RFS	0.99	0.92	1.06	0.710
CCND1	OS	1.21	0.83	1.76	0.321
CCND2	OS	0.77	0.63	0.95	0.014
CCND3	OS	0.69	0.42	1.14	0.145
Signature CCNDs	OS	0.88	0.76	1.01	0.071
CDK4	OS	1.55	0.84	2.84	0.161
MERC	OS	1.00	0.98	1.02	0.771
PKA Phosph. CREB	OS	1.00	0.94	1.07	0.930
BIOCARTA CREB	OS	0.96	0.91	1.03	0.245

Supplementary Table 4

Gene	Sequence
Human	
ND1 (mt.DNA) - Forward	CACCCAAGAACAGGGTTTGT
ND1 (mt.DNA) - Reverse	TGGCCATGGGTATGTTGTTAA
16S (mt.DNA) - Forward	CGAAAGGACAAGAGAAATAAGG
16S (mt.DNA) - Reverse	CTGTAAGTTTTAAGTTTTATGCG
18S (nucl.DNA) - Forward	ACGGACCAGAGCGCGAAAGCA
18S (nucl.DNA) - Reverse	GACATCTAAGGGCATCACAGAC
PUM1-Forward	GCTGCCGTGTTATCCAGAA
PUM1-Reverse	CCAGAGGCGTACAGGGATAG
POLMRT-Forward	ACGGAGACATCAGCCGAAAG
POLMRT-Reverse	GGCCCTTCCTGTAGCAGTG
TFB1M-Forward	CGGAAAACTCAGCACTTGCC
TFB1M-Reverse	ACAATCTTATCTGTCAGCCTCAAGT
TFB2M-Forward	ATGTCTTCTCGAGGGCTCTTT
TFB2M-Reverse	TTAAAGGGATGTCTGCTGTCCAA

Supplementary Table 5

Protein	Reference	Use	Dilution
CDK2	sc-163 (M2) (Santa Cruz)	WB	1/1000
CDK4	#1790 (D9G3E) (Cell Signaling Technology)	WB	1/1000
CDK6	#3136 (DCS83) (Cell Signaling Technology)	WB	1/2000
Cyclin D1	PA5-16607 (ThermoFisher)	WB	1/250
Cyclin D3	ab28283 [DCS2.2] (Abcam)	WB	1/1000
S780RB	#8180 (D59B7) (Cell Signaling Technology)	WB	1/1000
RB	sc-50 (C-15) (SantaCruz)	WB	1/1000
Cleaved Casp-3	#9664 (5A1E) (Cell Signaling Technology)	WB	1/1000
S616DRP1	#4494 (D9A1) (Cell Signaling Technology)	WB	1/1000
DRP1	#5391 (D8H5) (Cell Signaling Technology)	WB	1/1000
SERCA1	Ab129104 [EPR7322] (Abcam)	WB	1/1000
S1756ITPR1	#3760 (Cell Signaling)	WB	1/1000
ITPR1	07-1213 (MERCK) Lot#3546915	WB<br>PLA	1/1000<br>1/2000
ITPR2	sc-398434 (A-5) (Santa Cruz)	WB	1/1000
ITPR3	610313 (BD Tr.) Lot#2/IP3R-3	WB	1/1000
VDAC1	ab14734 (Abcam) Lot[20B12AF2]	WB<br>PLA	1/1000<br>1/1000
MCU	HPA016480-100UL (Sigma)	WB	1/1000
PKA Phospho-substrates	#9624 (100G7E) (Cell Signaling Technology)	WB	1/1000
CALR	#12238 (D3E6) XP® (Cell Signaling Technology)	WB	1/1000
SEC61B	#14648 (D5Q1W) (Cell Signaling Technology)	IF	1/200
CANX	10427-2-AP (LubioScience)	IF	1/200
PDH	Ab110334 (Abcam)	WB	1/1000
GRP75	#2816 (Cell Signaling Technology)	WB	1/1000
MFN2	#11925 (D1E9) (Cell Signaling Technology)	WB	1/1000
PRKAR1A	#5675 (D54D9) (Cell Signaling Technology)	WB	1/1000
TUB	T6199-200UL (Sigma)	WB	1/5000
ATP5A	ab14748 [15H4C4] (Abcam)	IF	1/500
PKACA	#4782 (Cell Signaling Technology)	IF	1/50
VAPB	15514012 (Invitrogen/Thermofischer)	PLA	1/500
PTPIP51	Orb101821 (BIORBYT Ltd)	PLA	1/1000
HA	#3724 HA-Tag (C29F4) (Cell Signaling Technology)	PLA	1/1000
Myc	#2276 Myc-Tag (9B11)) (Cell Signaling Technology)	PLA	1/1000
γH2AX	#2577 (Cell Signaling Technology)	IF	1/1000
BCL-xL	#2762 (Cell Signaling Technology)	WB	1/1000
BCL-2	#2876 (Cell Signaling Technology)	WB	1/1000
S112BAD	ab129192 [EPR1891(2)] (Abcam)	WB	1/500
BAD	#9292 (Cell Signaling Technology)	WB	1/1000
BAX	#2772 (Cell Signaling Technology)	WB	1/1000
Cytochrome C	#11940 (D18C7) (Cell Signaling Technology)	WB	1/1000

WB: Western Blot, IF: Immunofluorescence, PLA: Proximity Ligation Assay

Supplementary Figure 1: CDK4 is dispensable for TNBC tumor growth *in vitro* and *in vivo*
a-b Immunoblots and relative protein levels of CDK4, CDK6, CDK2, ^{S780}RB, $^{S807/S811}$RB, RB, Tubulin (TUB) and MEM code of CDK4-WT and CDK4-KO MDA-MB-231 TNBC cells, treated for 48 hours without (Veh) or with abemaciclib (Abema), or a combination abemaciclib + CDK2 inhibitor (Abema+AUZ). CDLK2inhibitor=AUZ=AUZ-454. N=2 independent biological replicates**.** 2-way ANOVA; Sidák's multiple comparison tests and genotype effects. **c** Immunoblots and relative protein levels of CDK4, Cyclin D1, Cyclin D3 and Tubulin, of CDK4-WT and -KO TNBC cells, N=3 independent biological replicates**. d** Venn diagram for RNA-seq data for genes differentially downregulated (log fold change <-0.5) or upregulated (log fold change> 0.5) and intersection with GSEA from cycling genes or E2F-target associated. **e-f** Representative tumor xenograft pictures and tumor xenograft volume at the sacrifice. N=8 WT and N=10 KO. Two-sided Unpaired T-test**. g** Tumor xenograft penetrance. N=20 WT and N=10 KO tumor xenografts. Two-sided Fisher's exact test. **h** Immunofluorescence of slices of CDK4-WT and -KO xenografts for Ki67 (red channel) and DAPI (blue channel). Representative pictures and associated quantification of Ki67 positive cells. Scale bars: 200 µm. N=8 WT and N=11 KO. Two-sided Unpaired T-test. Exact *p-values* are displayed in italic (bold italic if <0.05).

Supplementary Figure 2: CDK4 inhibition confers to TNBC cells resistance to chemotherapy *in vitro* and *in vivo*
a Percentage of viable cells compared of CDK4-WT and CDK4-KO MDA-MB-231 TNBC cells or pretreated with Vehicle or Abemaciclib (Abema) for 2 days , upon treatment with Vehicle, Cisplatin (10µM), 5-FU (20µM), or Doxorubicin (5µM) (Dox), H_20_2 (250µM-2hours), or Oligomycin and Antimycin (O+A) (1+10µM-2hours), UV_B (30mJ/cm^2) or TRAIL (2 µg/mL). N=3 biological replicates. 2-way ANOVA; Sidák's multiple comparison tests. **b** IC50 values for H_20_2 treatment in CDK4-WT and -KO TNBC cells. Representative of N=3 independent biological replicates. **c** Immunoblots of CDK4, Tubulin and MEM code number of CDK4-WT and different CDK4-KO clones (#10, #11 and #18), TNBC. **d** Quantification of the number of CDK4-WT and different CDK4-KO clones of TNBC cells, upon treatment with Vehicle, H_20_2 or O+A. N=4 biological replicates. 2-way ANOVA; Sidák's multiple comparison tests. **e I**ncreased percentage of viable cells for each treatment (Cisplatin, 5-FU, Doxorubicin (Dox), H_20_2 or O+A) from vehicle- to abemaciclib-treated cells. N=3 biological replicates. 2-way ANOVA; Sidák's multiple comparison tests. **f-g** Immunoblots and relative protein levels of ^{S780}RB, Tubulin (TUB), MEM code of triple-negative NST breast cancer patient-derived cells treated cells with Vehicle or Abema for 24 hours. N=3 biological replicates. Two-sided Unpaired T-test. **h** Immunoblots of ^{S780}RB, CDK4, TUB and MEM code of CDK4-WT and -KO MDA-MB-231 cell, HCC1806, BT-474, MDA-MB-168 and MCF-7 cells treated for 48 hours with Vehicle or Abema. **i** Number of viable MDA-MB-468 and MCF-7 cells, pretreated with Vehicle or Abema for 2 days and after treatment with Cisplatin (10µM), 5-FU (20µM),

or Doxorubicin (5µM for MDA-MB-468 and 10µM for MCF-7) (Dox). N=4 biological replicates. 2-way ANOVA; Sidák's multiple comparison tests. **j** Tumor volume of CDK4-WT and -KO tumor xenografts before the start of low Cisplatin (4mg/kg) treatment. 2-way ANOVA; Sidák's multiple comparison tests**.** **k** Tumor volume of CDK4-WT and -KO tumor xenografts before the start of high-Cisplatin (8mg/kg) treatment. 2-way ANOVA; Sidák's multiple comparison tests. **l** Tumor volume of CDK4-WT and -KO tumor xenografts, treated with vehicle (Veh) or high Cisplatin (8mg/kg). T=Treatment. N=4-5 mice per group. **m** Tumor volume of CDK4-WT and -KO tumor xenografts at sacrifice (D14). 2-way ANOVA; Sidák's multiple comparison tests. Mean +/- SEM of N=8 mice per group. **n** Representative CDK4-WT and -KO tumor xenografts pictures at sacrifice. **o** Immunoblots of CDK4, ^{S780}RB, Tubulin (TUB) and MEM code of CDK4-WT and -KO tumor xenografts, treated either with Vehicle or Cisplatin. N=4-5 mice per group. CDK4-WT and -KO tumor xenograft samples were loaded in two different gels, with subsequent same antibodies/exposure conditions. Exact *p-values* are displayed in italic (bold italic if <0.05).

Supplementary Figure 3: CDK4 regulates cell death modulating mitochondrial effectors of apoptosis in TNBC

a Immunoblots of CDK4, Tubulin (TUB) and MEM code of CDK4-WT, CDK4-KO TNBC cells transfected with empty plasmid HA (HA_Tr.) and CDK4-KO TNBC cells expressing endogenous CDK4 (CDK4_Tr.). **b-c** Representative pictures of DAPI staining and quantification of the number of CDK4-WT, CDK4-KO TNBC cells transfected with empty plasmid HA (HA_Tr.) and CDK4-KO TNBC cells expressing endogenous CDK4 (CDK4_Tr.), upon treatment with Vehicle, Cisplatin, H_20_2, or Oligomycin and Antimycin (O+A). Scale bars: 400µm. N=5-7 independent biological replicates. Mixed-Effect Analysis; Tukey's multiple comparisons test. **d-e** Representative pictures of DAPI staining and CDK4-WT and—KO MDA-MB-231 TNBC cells, upon treatment with Vehicle, Cisplatin, H_20_2, or Oligomycin and Antimycin (O+A). Scale bars: 200µm. N=5 independent biological replicates. 2-way ANOVA; Sidák's multiple comparison tests. **f-g** Representative pictures of DAPI staining and quantification of the number of viable pretreated cells with Vehicle (Veh) or Abemaciclib (Abema) for 8 days and after treatment with Vehicle, Cisplatin, H202, or Oligomycin and Antimycin (O+A). Scale bars: 400µm. N=5 independent biological replicates. 2-way ANOVA; Sidák's multiple comparison tests. **h** Immunoblots and relative protein levels of CDK4, ^{S780}RB, RB, Cleaved Caspase-3 (CL.CASP3), Tubulin (TUB) and MEM code of pretreated cells with Vehicle or Abema for 8 days and after treatment with Vehicle, Cisplatin, H202, or Oligomycin and O+A. Quantification of relative cleaved Caspase-3 protein levels (normalized to tubulin level). N=3 independent biological replicates. **i-j** Immunofluorescence of slices of CDK4-WT and -KO xenografts for Cleaved-Caspase 3 (green) and DAPI (blue). Representative pictures and associated quantification of Cleaved-Caspase 3 positive cells. 2-way ANOVA; Dunnett's T3 multiple comparisons tests. **k-l** Maximum amplitude/peak from basal mitochondrial calcium levels upon H_20_2

(2.5 mM) or O+A (100 µM, 10 µM) injections. N=4 independent biological replicates representing a total of n=135 cells for 10 independent injections (WT-H_2O_2), n=123 cells for 9 independent injections (KO-H_2O_2), n=128 cells for 9 independent injections (WT-O+A), n=128 cells for 9 independent injections (KO-O+A). Two-sided Paired T-tests. Exact *p-values* are displayed in italic (bold italic if <0.05).

Supplementary Figure 4: CDK4 participates in mitochondrial fission of TNBC

a Mitochondria perimeter of CDK4-WT and CDK4-KO TNBC cells according to electron micrographs. n=mitochondria. Representative of N=1 on 3 independent biological replicates. Two-sided Mann-Whitney Test. **b** Relative ratio of mitochondrial DNA (mtDNA) on nuclear DNA (nDNA). ND1 and 16S mitochondria-encoded genes and 18S nuclear-encoded gene. N=6 independent biological replicates. 2-way ANOVA; Sidák's multiple comparison tests. **c** Mitochondria perimeter of CDK4-WT and -KO tumor xenografts. n=mitochondria number accounting for 30 (CDK4-WT) and 41 (CDK4-KO) cells and from N=4 montages from 2 different tumor xenografts. Two-sided Unpaired T-test. **d** Mitochondrial number per cell of CDK4-WT and -KO TNBC cells according to electron micrographs (n=16 CDK4-WT cells and n=15 CDK4-KO cells). Representative of N=1 on 3 independent biological replicates. Two-sided Unpaired T-test. **e** GSEA Analysis for REACTOME_MITOCHONDRIAL_BIOGENESIS datasets from RNA-seq data on CDK4-WT and -KO TNBC cells. **f** Relative mRNA levels of *POLMRT*, *TFB1M* and *TFB2M* genes normalized to *PUM1* expression in CDK4-WT and -KO TNBC cells. N=3 independent biological replicates. Multiple two-sided Unpaired T-tests. **g** Mitochondrial aspect ratio (major axis/minor axis) and form factor (1/circularity) of CDK4-WT and -KO TNBC cells according to electron micrographs. n=mitochondria number representative from N=3 independent biological replicates. Representative of N=1 on 3 independent biological replicates. Two-sided Mann-Whitney Test. **h** Mitochondrial aspect ratio and form factor of CDK4-WT and -KO tumors xenografts. n=mitochondria number representative from 30 (CDK4-WT) and 41 (CDK4-KO) cells of N=2 tumor xenografts. Two-sided Unpaired T-tests **i** GSEA Analysis for GOBP_MITOCHONDRIAL_FUSION datasets from RNA-seq data on CDK4-WT and -KO TNBC cells. **j-k** Immunoblots and relative protein levels of ^{S780}RB, ^{S616}DRP1, DRP1 and Tubulin (TUB) and MEM code of TBNC cells pretreated cells with Vehicle or Abemaciclib (Abema) for 8 days. N=3 independent biological replicates. Two-sided Unpaired T-tests. **l** Representative pictures of mitochondria staining using Mitotracker of TBNC cells pretreated cells with Vehicle or Abema for 9 days. Associated quantification of mitochondrial aspect ratio and form factor. N=4 independent biological replicates representing of n=23 (CDK4-WT) and n=20 (CDK4-KO) cells. Two-sided Paired T-tests**.** Exact *p-values* are displayed in italic (bold italic if <0.05).

Supplementary Figure 5: CDK4 regulates ER-mitochondrial calcium signaling

a-b Maximum amplitude/peak and area under the curve (AUC) from basal mitochondrial calcium levels of CDK4-WT and CDK4-KO TNBC cells upon Thapsigargin (TG) (2 µM) or Histamine (Hist) (50 µM) injections. N=4 independent biological replicates representing a total of n=148 cells for 13 injections (WT-TG), n=142 cells for 13 injections (KO-TG), n=133 cells for 10 injections (WT-Hist.) and n=141 cells for 12 injections (KO-Hist.). Two-sided Paired T-tests. **c** Relative mitochondrial calcium levels of CDK4-WT and -KO TNBC cells upon Hist injections. Representative curve based on 4mtD3CPV fluorescence (Ratio YFP/CFP), normalized to baseline before injection. N=3 independent biological replicates. Arrow: time of injection. **d-e** Associated quantification of the AUC or maximum amplitude/peak of CDK4-WT and -KO TNBC cells upon Hist injections. N=12 injections, from 3 independent biological replicates representing a total of n=193 (WT-Hist.), n=229 cells (KO-Hist.). Two-sided Paired T-tests. **f-g** Maximum amplitude/peak and AUC from basal cytosolic calcium levels upon TG or Hist injections. N=3 independent biological replicates representing a total of n=107 cells for 9 injections (WT-TG), n=123 cells for 9 injections (KO-TG), n=121 cells for 9 injections (WT-Hist.) and n=118 cells for 9 injections (KO-Hist.). Two-sided Paired T-tests. **h** Maximum amplitude/peak from basal mitochondrial calcium levels of CDK4-WT, -KO TNBC cells transfected with empty plasmid HA (HA_Tr.) and -KO TNBC cells expressing endogenous CDK4 (CDK4_Tr.) upon TG treatment. N=6 injections accounting 2 biological replicates representing a total of n=75 cells (WT/HA_Tr.), n=55 cells (KO/HA_Tr.) and n=75 cells (KO/CDK4_Tr.). RM 1 way ANOVA; Tukey's multiple comparisons test. **i-j** Immunoblots and relative protein levels of CDK4, ^{S780}RB, ITPR2, ITPR3, VDAC1, MCU and Tubulin (TUB) and MEM code of CDK4-WT and -KO TNBC cells, pretreated with siCtrl, siITPR3, siVDAC1, siMCU, siITPR3/siVDAC1 or siVDAC1/siMCU. N=3 independent biological replicates. RM 1 way ANOVA; Tukey's multiple comparisons test. **k** Quantification of the number of CDK4-WT and -KO TNBC cells, pretreated with siCtrl, siITPR3, siVDAC1, siMCU, siITPR3/siVDAC1 or siVDAC1/siMCU. Three different independent loadings of the same samples were performed (#1: ITPR3 / #2: CDK4, ^{S780}RB, ITPR2, MCU and TUB / #3: VDAC1 and RB) with subsequent same antibodies/exposure conditions. N=3 independent biological replicates. 2-way ANOVA; Sidák's multiple comparison tests. *p-value* are indicated as numbers for this specific panel. Exact *p-values* are displayed in italic (bold italic if <0.05).

Supplementary Figure 6: CDK4 enhances Mitochondria-ER Contacts in TNBC

a Minimum ER-mitochondrion distance in analyzed MERCs**.** N=3 independent biological replicates representing n (CDK4-DWT) = 353 MERCs and n (CDK4-KO) = 351 MERCs. Two-sided Mann-Whitney Test. **b** Quantification of the number of MERCs per mitochondrion of CDK4-WT and -KO xenografts. Representatitve of N=2 independent xenograft samples representing a total of n (WT) = 44 cells and n

(KO) = 37 cells. Two-sided Mann-Whitney Test. **c** Representative electron micrographs of CDK4-WT and -KO tumor xenografts. Scale bars: 5µm. Red arrows indicate Mitochondria-ER Contacts (MERCs). **d** Representative micrographs of CDK4-WT and CDK4-KO TNBC cells immunolabeled with DAPI (blue), mitochondrial marker ATP5A (green), ER marker SEC61B (red). Quantification of thresholded Manders' coefficient (ATP5A-SEC61B) normalized to SEC61B staining. N=3 independent biological replicates accounting for n=56 cells (WT) and n=56 cells (KO). Two-sided Paired T-test. **e** Quantification of thresholded Manders' coefficient (ATP5A-CANX normalized to SEC61B staining of CDK4-WT and CDK4-KO TNBC cells. N=3 independent biological replicates accounting for n=33 cells (WT) and n=33 cells (KO). Two-sided Paired T-test. **f** Proximity Ligation Assay (PLA) using VAPB and PTPIP51 antibodies in CDK4-WT with different antibodies combinations. Associated quantification of VAPB-PTPIP51 dots per cell. N=4 independent biological replicates. Ordinary 1-Way ANOVA; Holm-Šídák's multiple comparisons test. **g** Proximity Ligation Assay (PLA) using VDAC1 and ITPR1 antibodies in CDK4-WT and -KO TNBC cells transfected with siCtrl or siVDAC1 with different antibodies combinations. Associated quantification of ITPR1-VDAC1 dots per cell. N=4 independent biological replicates. Ordinary 1-Way ANOVA; Holm-Šídák's multiple comparisons test. Exact *p-values* are displayed in italic (bold italic if <0.05).

Supplementary Figure 7: PKA activity is regulated by CDK4 but is not sufficient to mediate apoptosis

a Heatmap of InKA analysis for kinase activity. Code in CDK4-WT and CDK4-KO TNBC cells. N=4 independent biological replicates. **b** Phosphomotif and putative score of phosphorylation S83 by CDK4 of PRKAR1A and PRKAR1B from Phosphosite database**. C** Proximity Ligation Assay (PLA) using Myc and HA antibodies in CDK4-KO TNBC cells, previously transfected with CDK4-Myc, PRKAR1A-HA, PRKAR1B-HA plasmids. Quantification of Myc-HA dots per cell. PLA antibodies associations used are indicated below. N=6 biological replicates. Ordinary 1-Way ANOVA; Holm-Šídák's multiple comparisons test. Exact *p-values* are displayed in italic (bold italic if <0.05).

Supplementary Figure 8: MERCs-PKA activity is regulated by CDK4 and drives ER-MT calcium signaling

a Plot representing relative abundance of proteins detected though LC-MS/MS analyses in both whole cell lysate (WCL) and MERCs fractions of CDK4-WT TNBC cells. Student's T-test difference displays relative protein enrichment in MERCs fractions of CDK4-WT TNBC cells. **b** Volcano plot of phospho-proteomics data from the MERCs fraction of CDK4-WT and -KO TNBC cells. **c** Enrichment analysis on

phospho-peptides found in MERCS fraction of CDK4-WT and -KO TNBC cells. Benj. Hoch. FDR value ranges are displayed.

Supplementary Figure 9: CDK4 activity is positively correlated with apoptosis signature and better response to neoadjuvant chemotherapy (NAC) in TNBC patients.

a Dichotomized standardized survival curves showing recurrence free survival (RFS) probabilities in two subsets of patients according to their gene score expression based on median MERC signature, PKA Phosph. CREB score and BIOCARTA CREB score. 95% confidence intervals are displayed. SCAN-B TNBC patients treated with chemotherapy only. n=143 (High) and n=133 (Low) patients.

Supplementary Figure 10: CDK4 promotes mitochondrial fitness and metabolic flexibility through balanced calcium signaling

a Seahorse curves of oxygen consumption rates (OCR) from CDK4-WT and CDK4-KO MDA-MB-231 TNBC cells N=4 independent biological replicates. **b** Seahorse quantitative analysis of oxygen consumption rate (OCR). Basal OCR was evaluated in resting conditions, ATP-linked production OCR upon Oligomycin treatment, Maximal Respiration and proton leak OCR upon FCCP treatment. N=4 independent biological replicates. Multiple two-sided paired T-tests. **c** Energy phenotype profile determining oxygen consumption rates (OCR) in function of extracellular acidification rates (ECAR) of CDK4-WT and CDK4-KO TNBC cells in media containing Glucose (10 mM)/No galactose (Gluc+/Gal-), No glucose/Galactose (10 mM) (Gluc-/Gal+), or No glucose/No galactose (Gluc-/Gal-). Division of graph in 4 quarters were performed as following: Low OCR/Low ECAR = Quiescent, Low OCR/High ECAR = Glycolytic, High OCR/Low ECAR = Oxidative, High OCR/High ECAR = Mix Active. N=4 independent biological replicates. **d** Seahorse quantitative analysis of oxygen consumption rate (OCR) CDK4-WT and CDK4-KO TNBC cells in media containing Glucose (10 mM)/No galactose (Gluc+/Gal-), No glucose/Galactose (10 mM) (Gluc-/Gal+), or No glucose/No galactose (Gluc-/Gal-). Proton leak OCR was evaluated upon FCCP treatment. N=4 independent biological replicates. 2-way ANOVA; Tukey's multiple comparison tests. Exact *p-values* are displayed in italic (bold italic if <0.05).

Supplementary Figure 11: Graphical illustration summarizing the role of CDK4 in the regulation of mitochondrial-ER contacts.

The results suggest that CDK4 regulate the mitochondria-ER contacts through direct effects on both tethers and specific kinases, such as PKA, that regulate the transfer of calcium from the ER to the mitochondria. Under specific conditions, this increase in calcium is required to maintain mitochondrial dynamics, mitochondrial function, and apoptosis.

Supplementary Table 1

List of 176 MERC-associated proteins ranked by alphabetical order. Names are labeled according to gene nomenclature.

Supplementary Table 2

List of 141 detected MERC-associated proteins in the MERCs fraction of CDK4-WT and -KO MDA-MB-231 TNBC cells, ranked by alphabetical order. Described and main-associated function(s) of each protein is indicated. Names are labeled according to gene nomenclature.

Supplementary Table 3

Table of clinical dataset showing recurrence-free survival (RFS) probabilities in two subsets of patients according to their gene score expression based on median of Cyclin D1 (CCND1), Cyclin D2 (CCND2), Cyclin D3 (CCND3), signature of CCNDs, CDK4, Mitochondria-ER contact signature (MERC), PKA Phosph. CREB GSEA and BIOCARTE CREB GSEA. SCAN-B TNBC patients treated with chemotherapy only. RFS: Recurrence-free survival. OS: Overall survival. HR: Hazard ratio. Cox Proportional Hazards Survival Regression.

Supplementary Table 4

List of primers.

Supplementary Table 5

List of antibodies, including references, dilutions and applications. WB: Western Blot, IF: Immunofluorescence. PLA: Proximity Ligation Assay.

Supplementary Movie 1

Time-lapse acquisition of CDK4-WT MDA-MB-231 TNBC cells. Nanolive video constituted by 45 frames with one frame every 2 minutes.

Supplementary Movie 2

Time-lapse acquisition of CDK4-KO MDA-MB-231 TNBC cells. Nanolive video constituted by 45 frames with one frame every 2 minutes.
