# Supplementary material for: CDK4 inactivation inhibits apoptosis via mitochondria-ER contact remodeling in triple-negative breast cancer
Source: Nat Commun. 2025 Jan 9;16:541. doi: 10.1038/s41467-024-55605-z (PMC11718081; doi:10.1038/s41467-024-55605-z)
Supplement: Supplementary file 2 — Reporting Summary [file 41467_2024_55605_MOESM2_ESM.pdf]

nature portfolio

Corresponding author(s): LLuis Fajas

Last updated by author(s): 2024.12.16

Reporting Summary

Nature Portfolio wishes to improve the reproducibility of the work that we publish. This form provides structure for consistency and transparency in reporting. For further information on Nature Portfolio policies, see our Editorial Policies and the Editorial Policy Checklist.

Please do not complete any field with "not applicable" or n/a. Refer to the help text for what text to use if an item is not relevant to your study.
For final submission: please carefully check your responses for accuracy; you will not be able to make changes later.

Statistics

For all statistical analyses, confirm that the following items are present in the figure legend, table legend, main text, or Methods section.

n/a	Confirmed	
☐	☒	The exact sample size (n) for each experimental group/condition, given as a discrete number and unit of measurement
☐	☒	A statement on whether measurements were taken from distinct samples or whether the same sample was measured repeatedly
☐	☒	The statistical test(s) used AND whether they are one- or two-sided<br>*Only common tests should be described solely by name; describe more complex techniques in the Methods section.*
☐	☒	A description of all covariates tested
☐	☒	A description of any assumptions or corrections, such as tests of normality and adjustment for multiple comparisons
☐	☒	A full description of the statistical parameters including central tendency (e.g. means) or other basic estimates (e.g. regression coefficient) AND variation (e.g. standard deviation) or associated estimates of uncertainty (e.g. confidence intervals)
☐	☒	For null hypothesis testing, the test statistic (e.g. F, t, r) with confidence intervals, effect sizes, degrees of freedom and P value noted<br>*Give P values as exact values whenever suitable.*
☒	☐	For Bayesian analysis, information on the choice of priors and Markov chain Monte Carlo settings
☒	☐	For hierarchical and complex designs, identification of the appropriate level for tests and full reporting of outcomes
☐	☒	Estimates of effect sizes (e.g. Cohen's d, Pearson's r), indicating how they were calculated

Our web collection on statistics for biologists contains articles on many of the points above.

Software and code

Policy information about availability of computer code

Data collection	None
Data analysis	All data analysis are decribed in the "Methods" section

For manuscripts utilizing custom algorithms or software that are central to the research but not yet described in published literature, software must be made available to editors and reviewers. We strongly encourage code deposition in a community repository (e.g. GitHub). See the Nature Portfolio guidelines for submitting code & software for further information.

Data

Policy information about availability of data

All manuscripts must include a data availability statement. This statement should provide the following information, where applicable:

- Accession codes, unique identifiers, or web links for publicly available datasets
- A description of any restrictions on data availability
- For clinical datasets or third party data, please ensure that the statement adheres to our policy

Source data are provided with this paper. Generated datasets are publicly accessible as following: RNA-seq dataset (GSE278524) (https://www.ncbi.nlm.nih.gov/geo/query/acc.cgi?acc=GSE278524), proteomic and phosphoproteomic datasets (PXD046326, PXD046327 and PXD046353 on ProteomeXchange Consortium via the PRIDE partner repository 68) (respectively https://proteomecentral.proteomexchange.org/cgi/GetDataset?ID=PXD046326, https://proteomecentral.proteomexchange.org/cgi/GetDataset?ID=PXD046327, https://proteomecentral.proteomexchange.org/cgi/GetDataset?ID=PXD046353). The

neoadjuvant chemotherapies (NAC) and SCAN-B RNA-sequencing were downloaded from GEO using the accession code GSE12384539 and GSE60789 respectively. TCGA bulk RNA-sequencing (RNA-seq) datasets were downloaded from European Genome-Phenome Archive (http://www.ebi.ac.uk/ega/), under accession number EGAS00000000083. Any remaining data will be available from the corresponding author if needed.

Research involving human participants, their data, or biological material

Policy information about studies with human participants or human data. See also policy information about sex, gender (identity/presentation), and sexual orientation and race, ethnicity and racism.

Reporting on sex and gender	N/A
Reporting on race, ethnicity, or other socially relevant groupings	N/A
Population characteristics	N/A
Recruitment	N/A
Ethics oversight	N/A

Note that full information on the approval of the study protocol must also be provided in the manuscript.

Field-specific reporting

Please select the one below that is the best fit for your research. If you are not sure, read the appropriate sections before making your selection.

☒ Life sciences ☐ Behavioural & social sciences ☐ Ecological, evolutionary & environmental sciences

For a reference copy of the document with all sections, see nature.com/documents/nr-reporting-summary-flat.pdf

Life sciences study design

All studies must disclose on these points even when the disclosure is negative.

Sample size	Based on experience and G-Power software, sample sizes have been determined in order to have enough samples to detect statistical differences if any. For in vivo, this sample size is displayed as N=number of mice, usually designed as a mimimum of N=8 mice per group. These conducted in vivo experiments were approved by veterinary office of the canton of Vaud (Cantonal Licence #VD3726, National Licence #34118). For vitro, this number is determined as N=independent biological replicates in figure legends, from N=3 to N=6 were performed for each experiment. For vitro experiments, no sample calculation size was performed and biological replicates were performed with minimum N=3 replication.
Data exclusions	For data from from in vivo experiments, two-sided Grubb's test was performed to find outliers, which were removed from further analysis if the p-value was less than 0.05.
Replication	For in vivo data, experiments have been performed on independent littermate cohorts (N indicated in figure legends).<br>For in vitro data, we usually replicate experiments with 3 or 4 technical replicates, before proceeeding to a new independent biological replicate, displayed in the figures. For these data, all attemts of replication were successful and internal controls were fine.
Randomization	Allocation in the group was only determined for Cisplatin injection of CDK4-WT and CDK4-KO cells. A randomization was performed when tumor volume reached 80-120mm3. Mice were assigned in four different groups with equivalent mean tumor volume (+/-SD) and equivalent body weight (+/-SD) (Sup. Fig 2j-k). Apart from this experiment, we systematically used CDK4-WT or CDK4-KO TNBC cells and or +/- CDK4/6i-treated cells predetermined.
Blinding	For mice in vivo experiments, an identification code was given to each mice and samples. The corresponding genotype was checked only after performing experiments. For in vitro experiments, as mostly observational studies, we simply observed and recorded data thus making blinding not systematic. The mitochondria-ER contact analysis of electron micrographs was done blinded to avoid any measurement biais during the manual delination of organelles: pictures were randomely assigned with a number and the data collection was achieved afterwards.

Reporting for specific materials, systems and methods

We require information from authors about some types of materials, experimental systems and methods used in many studies. Here, indicate whether each material, system or method listed is relevant to your study. If you are not sure if a list item applies to your research, read the appropriate section before selecting a response.

Materials & experimental systems

n/a	Involved in the study
☐	☒ Antibodies
☐	☒ Eukaryotic cell lines
☒	☐ Palaeontology and archaeology
☐	☒ Animals and other organisms
☒	☐ Clinical data
☒	☐ Dual use research of concern
☒	☐ Plants

Methods

n/a	Involved in the study
☒	☐ ChIP-seq
☒	☐ Flow cytometry
☒	☐ MRI-based neuroimaging

Antibodies

Antibodies used

All primary antibodies are listed in Supplementary Table 5:
CDK2: sc-163 (M2) (Santa Cruz) WB use dilution 1/1000
CDK4: #1790 (D9G3E) (Cell Signaling Technology) WB use dilution 1/1000
CDK6: #3136 (DCS83) (Cell Signaling Technology) WB use dilution 1/2000
CyclinD1: PA5-16607 (ThermoFisher) WB use 1/250
CyclinD3: ab28283 [DCS2.2] (Abcam) WB use 1/1000
S780RB: #8180 (D59B7) (Cell Signaling Technology) WB use 1/1000
RB: sc-50 (C-15) (SantaCruz) WB use 1/1000
Cleaved Casp-3: #9664 (5A1E) (Cell Signaling Technology) WB use 1/1000
S616DRP1: #4494 (D9A1) (Cell Signaling Technology) WB use 1/1000
DRP1: #5391 (D8H5) (Cell Signaling Technology) WB use 1/1000
SERCA1: Ab129104 [EPR7322] (Abcam) WB use 1/1000
S1756ITPR1: #3760 (Cell Signaling) WB use 1/1000
ITPR1: 07-1213 (MERCK) Lot#3546915 WB use 1/1000, PLA use 1/2000
ITPR2: sc-398434 (A-5) (Santa Cruz) WB use 1/1000
ITPR3: 610313 (BD Tr.) Lot#2/IP3R-3 WB use 1/1000
VDAC1: ab14734 (Abcam) Lot[20B12AF2] WB and PLA use 1/1000
MCU: HPA016480-100UL (Sigma) WB use 1/1000
PKA Phospho-substrates: #9624 (100G7E) (Cell Signaling Technology) WB use 1/1000
CALR: #12238 (D3E6) XP® (Cell Signaling Technology) WB use 1/1000
SEC61B: #14648 (D5Q1W) (Cell Signaling Technology) IF use 1/200
CANX: 10427-2-AP (LubioScience) IF use 1/200
PDH: Ab110334 (Abcam) WB use 1/1000
GRP75: #2816 (Cell Signaling Technology) WB use 1/1000
MFN2: #11925 (D1E9) (Cell Signaling Technology) WB use 1/1000
PRKAR1A: #5675 (D54D9) (Cell Signaling Technology) WB use 1/1000
TUB: T6199-200UL (Sigma) WB use 1/5000
ATP5A: ab14748 [15H4C4] (Abcam) IF use 1/500
PKACA: #4782 (Cell Signaling Technology) IF use 1/50
VAPB: 15514012 (Invitrogen/Thermofischer) PLA use 1/500
PTPIP51: Orb101821 (BIORBYT Ltd) PLA use 1/1000
HA: #3724 HA-Tag (C29F4) (Cell Signaling Technology) PLA use 1/1000
Myc: #2276 Myc-Tag (9B11)) (Cell Signaling Technology) PLA use 1/1000
γH2AX: #2577 (Cell Signaling Technology) IF use 1/1000
BCL-xL: #2762 (Cell Signaling Technology) WB use 1/1000
BCL-2: #2876 (Cell Signaling Technology) WB use 1/1000
S112BAD: ab129192 [EPR1891(2)] (Abcam) WB use 1/500
BAD: #9292 (Cell Signaling Technology) WB use 1/1000
BAX: #2772 (Cell Signaling Technology) WB use 1/1000
Cytochrome c: #11940 (D18C7) (Cell Signaling Technology) WB use 1/1000

Validation

- CDK2, CDK6, RB, DRP1, HA, MYC: validation according manufacturer's website through overexpression and bsed on expected size of the bands.
- CDK4: validation by our CDK4-KO model in this manuscript Fig 1A, based on expected size of the band
- Cyclin D3, PDH, VDAC1: KO or RNAi validation according manufacturer's website, based on expected size of the bands.
- S780RB: validation according manufcaturer's website, based on expected size of the band using serum.
- Cleaved Caspase-3, S616 DRP1, PKA phosphosubtrates, γH2AX, BCL-xL, BCL-2, S112BAD, BAD, BAX, Cytochrome c: validation according manufacturer's website through use of specific treatment (e.g. pro-apoptotic treatment).
- SERCA1, ITPR1, ITPR2. ITPR3, S1756ITPR1, CALR, GRP75, PRKAR1A, TUB, ATP5A, PKACA, VAPB, PTPIP51: validation according manufacturer's website based on expected size of the bands, and also routinely tested.

Eukaryotic cell lines

Policy information about cell lines and Sex and Gender in Research

Cell line source(s)

TNBC MDA-MB-231, BT-474, HCC1806, MDA-MB-468, and ER+ PR+ MCF7 are from ATCC. Triple-negative NST breast cancer

Cell line source(s)	luciferase-expressing cells were dissected and biable frozen from a xenografted primary tumor derived from female patient 10 (T70), as described in (Sflomos et al., 2006).
Authentication	Cells were amplified and frozen upon receipt from the company before their use. No further authentifications were performed.
Mycoplasma contamination	All cell lines were checked and tested negative for mycoplasma contamination. Patient-derived xenograft NST breast cancer cells were not tested for mycoplasma contamination.
Commonly misidentified lines (See ICLAC register)	No commonly misidentified cell lines were used in this study.

Animals and other research organisms

Policy information about studies involving animals; ARRIVE guidelines recommended for reporting animal research, and Sex and Gender in Research

Laboratory animals	NOD SCID gamma (NSG) mice from The Jackson Laboratory were bred at the animal facility specific pathogen-free of the University of Lausanne, Epalinges. Acclimatation of two weeks was done with general monitoring before starting mammary fat pad injection. Mice were maintained in laminar-flow boxes under standard conditions (standard diet and water ad libitum) at 23°C, 50% hygrometry, with 12 hrs light and 12 hours dark cycles in Center for Integrative Genomics main animal facility. 2 x 106 MDA-MB-231 CDK4-WT or -KO cells in 50µL of 1X PBS were injected into the fat pad of the 4th mammary gland of 8-week-old females. The experiments were approved by the Direction générale de l'agriculture, de la viticulture et des affaires vétérinaires (DGAV) (Cantonal License #VD3726, National License #34118) according to art. 18 Animal Welfare Act (SR 455), art. 141 Animal Welfare Ordinance (SR 455.1), art. 30 Animal Experimentation Ordinance (SR 455.163). The conducted methods were in accordance with the animal care guidelines of Swiss laws, following 3R recommendations.
Wild animals	This study did not involve wild animals.
Reporting on sex	Sex was considered in this study as breast cancer mainly (99.99%) affect women, female mice were then naturally used for the vivo experiment.
Field-collected samples	No field-collected samples were used in this study.
Ethics oversight	Protocols and sample sizes, determined in order to have enough samples to detect statistical differences, if any, were approved by the Direction générale de l'agriculture, de la viticulture et des affaires vétérinaires (DGAV) (Cantonal License #VD3726, National License #34118).

Note that full information on the approval of the study protocol must also be provided in the manuscript.

Plants

Seed stocks	N/A
Novel plant genotypes	N/A
Authentication	N/A

This checklist template is licensed under a Creative Commons Attribution 4.0 International License, which permits use, sharing, adaptation, distribution and reproduction in any medium or format, as long as you give appropriate credit to the original author(s) and the source, provide a link to the Creative Commons license, and indicate if changes were made. The images or other third party material in this article are included in the article's Creative Commons license, unless indicated otherwise in a credit line to the material. If material is not included in the article's Creative Commons license and your intended use is not permitted by statutory regulation or exceeds the permitted use, you will need to obtain permission directly from the copyright holder. To view a copy of this license, visit http://creativecommons.org/licenses/by/4.0/
