# Supplementary material for: CDK4 inactivation inhibits apoptosis via mitochondria-ER contact remodeling in triple-negative breast cancer
Source: Nat Commun. 2025 Jan 9;16:541. doi: 10.1038/s41467-024-55605-z (PMC11718081; doi:10.1038/s41467-024-55605-z)
Supplement: Supplementary file 3 — Description of Additional Supplementary Files [file 41467_2024_55605_MOESM3_ESM.pdf]

Supplementary Movie 1

Time-lapse acquisition of CDK4-WT MDA-MB-231 TNBC cells. Nanolive video constituted by 45 frames with one frame every 2 minutes.

Supplementary Movie 2

Time-lapse acquisition of CDK4-KO MDA-MB-231 TNBC cells. Nanolive video constituted by 45 frames with one frame every 2 minutes.
